# What Counts as ‘Real’ in Healthcare Interaction? Examining Human/Technology Hybrid Simulation Interactions in Communication Skills Training

**DOI:** 10.1111/1467-9566.70284

**Published:** 2026-10-19

**Authors:** Alison Pilnick, Lauren Bridgstock, Saul Albert, Rebecca O'Brien, Anne‐Marie Borneuf

**Affiliations:** ^1^ School of Nursing and Public Health Manchester Metropolitan University Manchester UK; ^2^ Communication and Media, School of Social Sciences and Humanities Loughborough University Loughborough UK; ^3^ School of Health Sciences University of Nottingham Nottingham UK; ^4^ Nottinghamshire Healthcare NHS Foundation Trust Nottingham UK

## Abstract

Simulated interaction is a common healthcare training tool and is increasingly technologically mediated. Although the need for *clinical* realism or authenticity of simulated interactions is commonly understood, less attention has been paid to their *interactional* realism. However, research beyond healthcare suggests that participants interact differently in simulated scenarios. This paper examines simulations conducted through the MetaHuman Live platform, where a human tutor is live‐linked to an avatar using the tutor's voice and mapping their gaze and facial expressions. We use conversation analysis (CA) to analyse student nurses' interactions with this platform, identifying key areas where they differ from ‘real’ interactions. These are openings, closings, establishing shared knowledge, interactional progressivity and orientation to simulation platforms as an assessment tool. We suggest that features of a hybrid platform which aim to increase realism can also make interaction more complicated for learners trying to navigate between ‘the person’ and ‘the system’. We conclude that, although our analysis suggests ways forward for the more effective integration of simulation technologies in training communication skills in healthcare, it also points to our continued need for a better sociological understanding of how interaction itself works in practice, to provide an evidential basis for design and evaluation of such simulations.

## 1 Introduction

Communication is the most common subject of all new complaints made to the NHS about hospital and community health services (NHS Digital 2024). Communication skills training (CST) is a critical workforce development and retention issue for the NHS, specifically referenced in the national core standards for healthcare professionals (HCPs) in England (Health and Care Professions Council 2023). HCPs often report lacking confidence and needing more training, particularly for groups and settings that are commonly experienced as ‘challenging’, such as breaking bad news, managing conflict or caring for people living with dementia (e.g., Griffiths et al. 2014). However, evidence suggests that communication skills cannot be improved through clinical experience alone (Bylund et al. 2022).

One common way of providing CST is by using simulated scenarios, where patients are portrayed by clinical tutors, specialist actors or increasingly, by avatars in technologically mediated communication simulation platforms. Although research on simulation tends to evaluate learner experience and satisfaction rather than the conduct of the simulated interaction itself, a substantial body of work does assess the effectiveness of simulation‐based communication training, including through systematic and scoping reviews and through approaches that ground training in conversation analytic findings (Pilnick et al. 2018, 2023; Albert et al. 2025; L. B., A. P., N. Hammond et al., Unpublished data). This body of research suggests that once learners overcome initial apprehensiveness, they experience simulation as a useful teaching experience, because of the need to think and respond in real time and the ability both to receive feedback on performance and to observe and reflect on how others approach simulation tasks. The design and marketing of technologically mediated simulation platforms aimed at HCPs often emphasise their *clinical authenticity*, in terms of the clinical realism of the scenarios they depict (see Guraya 2024; Lavoie et al. 2020). However, relatively little is known about the effectiveness of simulation in teaching the authentic communication skills that HCPs need to develop as part of their clinical practice, or the specific impacts of simulation technologies on the communication training process. Responding to this need, this paper examines video recordings of student nurses using a technologically mediated communication training simulation platform: MetaHuman Live (MHL), focusing on the interactional conduct of these simulations. We conclude by considering the implications for how these systems are embedded in the training of HCPs. We also reflect on what this means for our wider understanding of how human interaction in healthcare is reshaped when it is technologically mediated and simulated.

## 2 Background

Simulation training for HCP learners has traditionally involved role play with mannequins, other HCP learners or tutors or the use of specially trained actors known as simulated patients (SPs). Despite the fact that healthcare simulation is an interactional process, there has to date been little sociological scrutiny of these kinds of interactions or of the impact of technological mediation. Broader sociological considerations have largely been limited to overviews which highlight the legal, ethical and social implications of these technologies, such as the potential for underpinning language models to cause linguistic exclusion (Rudschies and Schneider 2024). However, those studies which do exist have pointed to the importance of the ‘infidelity of place’ (Bartram 2020) or of ‘frame shifting’ (Iedema et al. 2009) in the learning process: the idea that certain liberties from reality need to be taken during simulation so that the tutor can help the learner to prepare better for a future clinical reality. Despite these claims, the features that differentiate ‘training’ talk from ‘realistic’ talk in this context, and how participants can or should move between them, are poorly understood.

Although it is perhaps unsurprising that a learner might interact differently with a mannequin than with a real person, more detailed interactional studies of communication in simulated environments also suggest that learners interact differently in simulated scenarios with human actors than with real patients (Atkins 2019). This can lead to unreliable evaluations of competence, which have consequences for both practitioners and patients (Stokoe et al. (2020); Atkins et al. 2016).

Technologically mediated simulations have varying degrees of sophistication, with some employing artificial intelligence (AI) as a stand‐alone method of generating talk or text, and others requiring control by a human participant. User interfaces also vary: some platforms involve the trainee typing or selecting predefined responses, whereas others allow some method of spoken response. This variability in technological implementation produces a practical difficulty of description and categorisation, even before considering the impact of these differences on interaction. This means that ‘techniques and technologies that travel under the sign of AI’ (Suchman 2023, 1) cannot be treated as an unproblematic or unitary category. Brooker et al. (2019, 296) underline the need to ‘describe how AI features in the world as it is’: how these technologies are put to practical use with human participants. Because most evaluations rely on post hoc self‐report, they may miss the authentic details of interaction that are not usually accessible for participants' introspection, such as pauses, disfluencies or overlapping talk. As Albert et al. (2025) note, even when experts set out to simulate an interaction, they may draw on intuitive assumptions that do not correspond with recordings of real interactions. We draw here on the burgeoning ethnomethodological and conversation analytic (EMCA) literature on AI in situated interaction (see Mlynář et al. 2024) and use conversation analysis (CA) to discover features of these simulations that may not be intuitively imagined or reported on (Sacks 1984, 25).

Brandt et al. (2024), in their work on the development of a healthcare chatbot, explain how an EMCA approach to social interaction demonstrates the highly systematic structure of talk‐in‐interaction and enables understanding of how engagements with AI systems differ from human–human interactions. In interactions between humans, speakers monitor each other's behaviours to tailor their contributions in an appropriate and timely manner. However, in a hybrid human–computer system, human participants may be unsure what conversational competence to attribute to their nonhuman coparticipant. Although the agent of action in the field of human–computer interaction (HCI) studies is usually conceptualised as ‘a contrastive binary of either human or computer’ (Albert and Hall 2024, 905), this distinction is not necessarily relevant to the interactional practices involved. An empirical focus on the interaction itself, rather than on technological features or the imagined ‘authentic’ characteristics of human participants, can help to avoid these categorical complexities of HCI studies of simulation training. This focus is particularly pertinent in the case of MetaHumans Live (MHL) (Epic Games 2025): a form of technologically mediated simulation which uses a virtual avatar or ‘MetaHuman’, live‐linked to a human simulator. The avatar's facial features are mapped onto the human operator's face, so it replicates patterns of movement, gaze and facial expression produced by the live‐linked human. Participants can talk to the avatar through a video platform such as Microsoft Teams, and the avatar's appearance can be varied by sex, ethnicity and age. Although computer‐generated speech is often criticised for lacking the ostensibly ‘messy’ yet systematic patterns of gaps, overlaps and perturbations (Stokoe et al. 2020), the authentic voice of the MHL avatar is produced in real time by a human operator.

When speaking with virtual agents and other computer‐generated voice interfaces, human participants tend to adapt to the limitations of the speech recognition system, for example by speaking more slowly and with simpler syntax (Pelikan and Broth 2016). The assumption underlying the MHL approach to simulation is that a real human simulator with an avatar that can take on different physical appearances will produce more convincing and varied simulations. However, as our analysis will show, this hybrid approach to human/computer simulation may actually create additional difficulties for interactants.

We ground this analysis in two empirical questions. First, to what extent do the simulator and the trainee interact in ways that are recognisable from empirical studies of everyday healthcare settings such as telephone‐mediated delivery of clinical news to relatives, and from what we know about how these vary across healthcare contexts in routine, emergency, primary or secondary care (Parry and Barnes 2024). Second, to what extent, if at all, do participants orient to the technologically mediated and simulated nature of the environment, and if so, does this disrupt their interactional patterns? Following Reeves (2019), our interest is in how these issues are consequential for the organisation of social interaction such as turn‐taking procedures, lexical choice or dealing with troubles of speaking, hearing and understanding.

## 3 Methods

The study was conducted with 10 nursing students aged 19–52 at a UK university. The participants were seven females and three males; nine reported that English was their first language, with one reporting Arabic. Fluency in written and spoken English was a requirement to be accepted onto their degree courses. Eligible participants were over the age of 18 and had prior experience of simulation training using the standard MHL platform during their course, so that this would not be their first experience of technologically mediated simulation. This meant that all students were in Year 2 or above of nursing training, and would have already completed some simulated communication skills training. Participants were recruited through poster advertisement and relevant university email mailing lists. Ethical approval for this study was granted by the Health and Education Research Ethics and Governance Committee at Manchester Metropolitan University (reference number 67498). These simulations were conducted solely for research purposes, to generate naturalistic data on how trainees interact with the MHL platform. Participants were told that their performance would not be formally assessed and that our interest was in how people interacted with the technology.

For the purpose of this study, an MHL avatar was custom‐edited to represent a woman in her 50s sitting in a home environment. The avatar was named ‘Michelle’ and was represented as having the same ethnicity as the tutor who was live‐linked to her, though we did not seek to directly represent other aspects of the tutor's appearance or actual age for the scenario. The live‐linked tutor was an experienced clinical tutor with particular expertise in simulation training. She was asked to conduct the simulation as she would ordinarily conduct a simulation exercise. Figure 1 shows a screenshot of the avatar and the background that was visible to participants.

**FIGURE 1:**
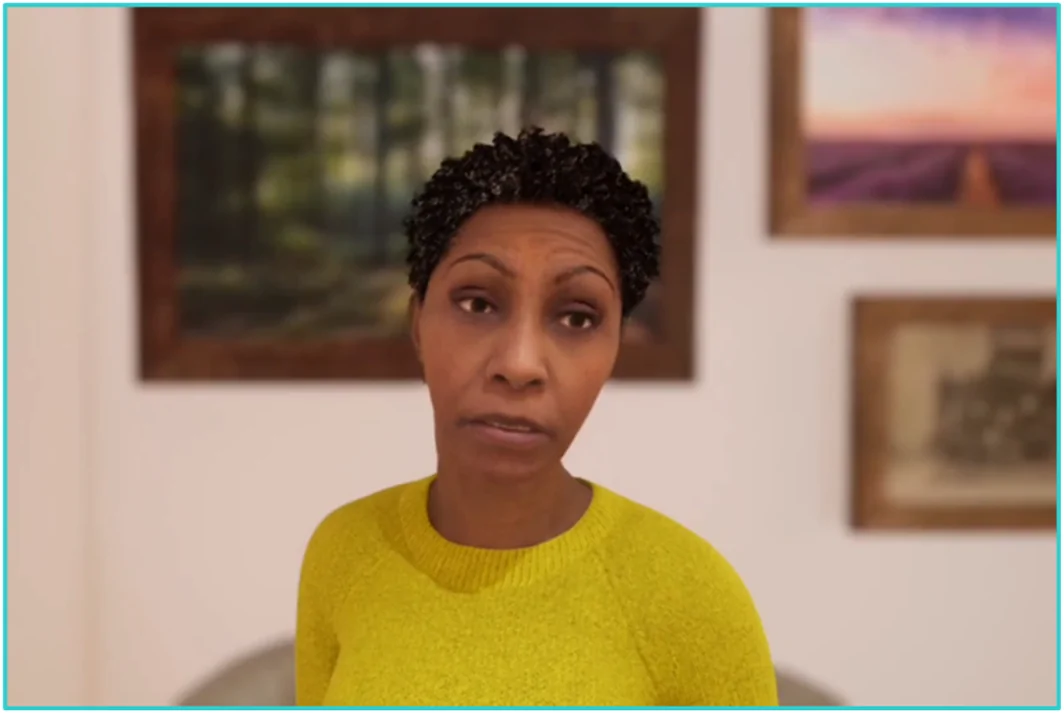
‘Michelle’.

Before the simulation, participants were given the following scenario on a piece of paper: ‘Dave is a 50‐year‐old man who has been brought into the emergency dept at 9 a.m. by ambulance following a cardiac arrest at home. He came in on his own. Whilst in the ED, he was successfully resuscitated but is critically ill. He has an endotracheal tube in place to support his breathing using a ventilator, and he is sedated. He has been assessed by the ICU team and is waiting for a bed to be free so that he can be transferred to cardiac ICU. You need to phone his wife, Michelle, to let her know what has happened.’ Participants were then shown to a laptop in a separate room and asked to make a Microsoft Teams call to Michelle when they were ready to start the simulation.

During the simulation, participants were video recorded, using both the internal Teams recording feature and a stand‐alone video camera placed in the room where they made the call. Given the importance of gaze in social interaction (Rossano 2012), the ability of MHL to live‐link human gaze is proposed as a particularly significant feature of the platform, and we therefore wanted to be able to see where a participant's gaze was directed when it was not directed at the screen.

Recordings were then analysed using conversation analysis. CA is a sociological method which has been widely used to analyse healthcare communication in other contexts (see Parry and Barnes 2024). In accordance with the guidance set out by Sidnell (2013), analysis involved three stages: observation of the data as a whole (transcribed according to Jefferson's (2004) conventions); identification of relevant phenomena; and collection of examples resulting in descriptions of practices built up from both single examples and comparisons across multiple examples. Data analysis sessions took place with the project team. After repeated viewing and transcription of the full corpus, candidate phenomena were identified inductively as recurrent, interactionally consequential features of the data (e.g., disordered openings or the verbatim reading of the scenario brief). For each phenomenon, a ‘collection’ of instances was assembled across the ten recordings, and individual cases were analysed turn by turn, compared with one another and with established CA findings on related practices in naturally occurring talk (Clayman 2024). Analytic claims were grounded in participants' own displayed orientations, that is, to how each next turn treats the prior turn.

Once the simulations were complete, a subset of participants also took part in a focus group to discuss their experiences. We draw on the focus group only for a small number of points below where participants' post hoc accounts help to illuminate the interactional phenomena in question; focus group findings are discussed in more detail in a separate paper (Bridgstock et al., forthcoming).

## 4 Analysis

Across the ten simulations, interaction departed from the normative patterns documented in comparable situations in naturally occurring talk in several significant ways. Mlynář et al. (2024) found that existing studies of AI as situated action tended to centre on three key themes: opening and closing of interactions; miscommunication; and nonverbal aspects of interaction. It is therefore unsurprising that we also found issues relating to these three themes in our data, though the ways in which they manifested were often specifically related to the hybridity of the platform. We focus our analysis here on four phenomena: openings; establishing what is treated as shared knowledge; progressivity of the interaction; and closings. Openings in our data were often marked by interactional trouble, and management of ostensibly shared knowledge was hampered by reference to the training scenario brief. In some cases, the interactions did not display markers of urgency or concern where it might be expected, and closings were often truncated or entirely absent. In all cases, there was evidence that participants oriented to the platform as an assessment tool. In the analysis below, we develop these findings in detail. We begin by considering openings.

### 4.1 Openings

As Brandt et al. (2024) describe, the routine nature of telephone call openings makes them a particularly fruitful environment for studies of technologically mediated interaction. When initiating a call, a range of actions may be relevant (such as greetings, identification sequences and providing the reason for the call). What callers and recipients choose to include or leave out is a potential analytic resource, because it shows their understandings of what kind of call they are engaged in. For example, the ‘how are you’ sequences that are common in telephone calls between friends and family are typically absent from institutional calls (Whalen and Zimmerman 1987). In the case of an unsolicited and unexpected call from an unknown healthcare professional (as is the case here), the caller (the student participant) needs to convince the call recipient (the patient's wife) that they are a legitimate caller with a legitimate purpose. In calls of this kind, recipients do not necessarily disengage simply because it is a ‘cold call’. Instead, establishing the caller's legitimacy and purpose is the interactional work that is normatively due early in the call, and difficulty in accomplishing and ratifying this ‘reason for the call’ can make the subsequent business of the call harder to transact (Stokoe et al. 2020).

Decades of CA research have shown that the standard response to the summons of a ringing phone is an answer sequence which provides some kind of acknowledgement or greeting. Schegloff (1968, 1076) describes how, in the absence of caller ID, the answerer of the call typically speaks first on picking up the receiver and the caller then replies with a greeting and often some sort of continuer such as a term of address or self‐identifier (depending on familiarity). However, in the majority of our recordings, the caller was the participant who spoke first on the call. This is despite the fact that, in this simulation, the student initiates the call by pressing the call button (which generates a calling sound), and the tutor portraying the relative then clicks to accept this call. Although this process of simulation closely mimics the ordinary process of making and receiving a phone call, and in this scenario the participants have additional visual cues that the call has connected, the participants then appear confused as to who should do the interactional work of initiating the call. The extract below is an example of this^1^:


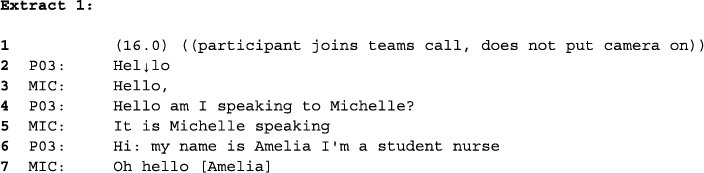


In Extract 1, after a 16‐second pause, it is the student participant who initiates the call, with a greeting which is reciprocated by Michelle. However, the learner then produces an additional greeting and an identification at line 4, of the kind that would normally be the first utterance of a caller. Just as callers sometimes issue a ‘second summons’ in call openings where there is trouble with the audio or with mutual identification (Schegloff 1968; Lee 2006), in this case the learner ‘redoes’ the call opening utterance, resulting in a second round of orderly greetings and mutual introductions from which the call proceeds. A similar opening pattern is seen in Extract 2:


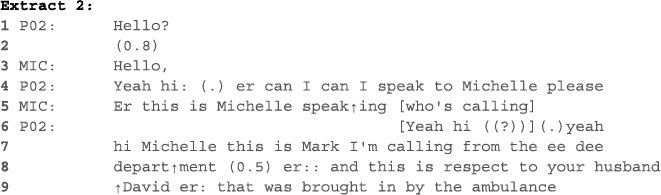


In this extract, the learner once again initiates the interaction, and his subsequent utterance ‘Yeah hi’ attends to the repetition of his greeting in Line 4. Brandt et al. (2024) describe how, when normative patterns for call openings are breached, participants are unable to pursue the normative trajectory for the activity and ‘have to actively work at discovering the order for this interaction simulation’ (2024, 910). As here, this discovery may also necessitate additional interactional work in order to restore the progressivity of the call.

On the rare occasions in our data where Michelle did make the first utterance, there was some kind of nonlexical vocalisation giving an indication that the participant was present prior to Michelle initiating a greeting, as is the case in Extract 3:


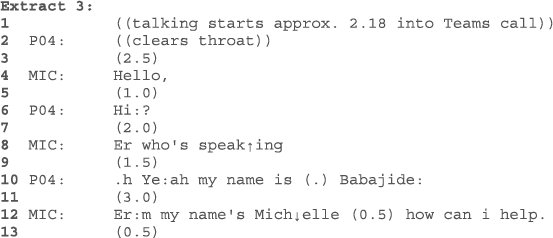


In Extract 3, there is silence for over 2 minutes after the call is connected. Then, on line 2, P04 makes a sound like they are clearing their throat, prompting Michelle to initiate a greeting with a rising intonation, treating the sound as evidence of the other participant's presence. When Michelle speaks in line 4, there is another pause before the student participant begins talking on line 6. This protracted opening sequence, and the student participant initially offering only a greeting, suggests some confusion over who should move the interaction on to the reason for the call (and the business of the simulation).

Although we have used the literature on telephone calls in setting up our analysis here, it seems at least probable that part of the ambiguity in our data is produced by the mutual visibility that is available at the start of the call. Licoppe's (2017) work on personal Skype calls showed how openings can be much more variable than typical phone call opening sequences because of factors including irregularities in the order of audio and video appearance, meaning both the caller and the recipient may be the first speaker. Licoppe (2017) further noted that becoming visible and ‘appearing’ are different things, as in his data, participants wait to greet the other person on the call until they ‘appear’ by moving closer and gazing at the screen, despite their image appearing prior to this as soon as the call connects. Overall, Licoppe concludes that participants in the video calls use recognising or noticing an ‘appearance’ as an interactional resource for achieving a joint frame of interaction, and this impacts on whether the caller is the person to speak first. Certainly, our video footage gained from the room in which the student callers sat suggests there was an orientation to ensuring features of the technology were working correctly before they began to speak. However, this disruption to opening sequences also has practical implications in this hypothetical institutional context, because delays in initiation sequences in turn create delays in attending to the business of the call, where the student needs to deliver the information they have been tasked with delivering.

Stokoe et al. (2020) have highlighted the way in which CA research across both institutional and ordinary conversational settings has shown how callers articulate a ‘reason for the call’ (Sacks, 1992) in or near their first speaking turn, after greetings and identification. Here, however, we see that this norm is also disrupted; Extract 4 shows an example of this delayed production, which is eventually prompted by Michelle's request in Line 10.


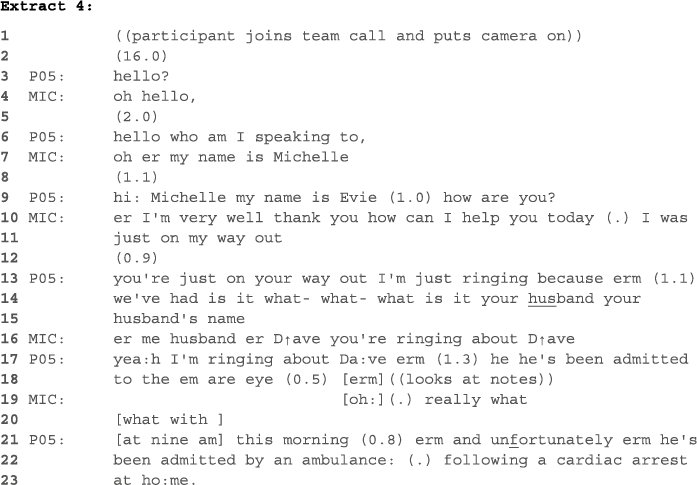


In this extract, there is a substantial delay before P05 begins speaking (line 3). Michelle responds with a greeting, and after a pause P05 produces another greeting, followed by a request for the call recipient to identify herself. Although it is usual in healthcare encounters, because of the confidential nature of the information involved, to verify that a healthcare professional is speaking to the correct person, it is less usual to put the onus on a call recipient in this way in the absence of the caller first establishing their institutional identity. However, Michelle does provide a response, and after a pause P05 embarks on a third greeting sequence followed by a ‘how are you’ question. This question is unusual in institutional calls (Whalen and Zimmerman 1987) and perhaps particularly unusual in those characterised by urgency. Michelle responds to this enquiry, but then immediately asks how she can help, thereby indirectly eliciting the reason for the call. P05 subsequently does begin to outline this, although a clear reason is not explicitly stated until lines 17–18, being further delayed by a sequence to establish Michelle's husband's name before giving details of his admission to hospital. (‘Em are eye’ (MRI) here refers to a common abbreviation for a local hospital). The potential risk of such a protracted sequence and such a delayed production is obvious; if this were an actual interaction to inform a patient's relative of urgent news, the call may already have been terminated before this point can be reached.

### 4.2 Reading From the Paper: The Problem of Relevant (Shared) Knowledge

Having considered how these calls are opened, we now turn to consider what the participants treat as ‘known’ during their interaction. In our data, the availability of a ‘script’ in the form of the scenario brief and its relevance within the interaction also lead to problems of epistemics, that is, how participants' relative rights to knowledge are displayed and managed interactionally (Heritage and Raymond 2005, 2012). For example, the student making the call must work out how to deliver Dave's cardiac arrest as ‘news’ for the other participant, and the simulator has to work out how to receive it, interactionally, as news, despite the fact that both parties know that the simulator knows the call is coming. As we see in Extract 5, producing news delivery or news receipts and dealing with who knows (and says) what in this setting can be hard to simulate.


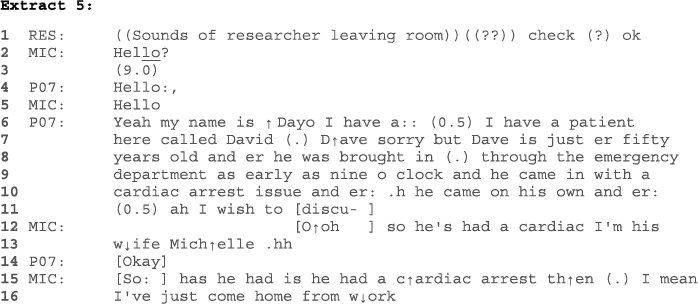


In this example, the participant begins the call as the researcher was leaving the room, so Michelle initiates a greeting after hearing this noise. After greetings and self‐identification, the student embarks immediately on describing the specifics of the situation with Dave, though he does not say where he is purportedly calling from. During his turn from lines 6–11, he repeats much of the information given on the scenario paper word for word, including the term ‘cardiac arrest’ and other information from the scenario outline such as the patient's precise time of arrival at the hospital. He uses details from the scenario paper to inform Michelle of things she would obviously know if she were Dave's wife, such as Dave's age. In all the interactions in our data, the student participant used phrases and terms taken directly from the scenario briefing which were ill‐fitted to the unfolding interaction either in relevance or general intelligibility. In some cases, this was picked out by the simulator. For example, in Extract 6 below, Michelle asks about the meaning of the term ‘cardiac arrest’^2^.


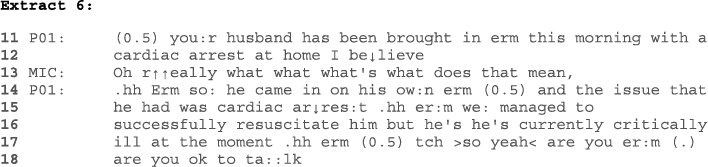


Although Michelle asks for an explanation in line 13, in lines 14–15 the student simply repeats ‘cardiac arrest’ without elaborating on its meaning, instead continuing by providing more details from the scenario outline that have no obvious immediate interactional relevance, such as that Dave came into hospital on his own. The precise time of arrival in the hospital, and the fact that Dave arrived alone, were recurrently included by student nurses in these interactions. They also commonly used other technical terms that were given in the scenario brief (such as ‘endotracheal tube’) without any further explanation. We do not assume that such terms are necessarily unknown to relatives, whose familiarity with clinical language will vary widely. Our interactional point here is that where the recipient treats a term as problematic, as Michelle does here with ‘cardiac arrest’, students nonetheless persist with the wording as it appears in the brief rather than reformulating it for this particular recipient. As in the example above, these terms were repeated even when they were problematised by Michelle. This verbal reliance on the exact text provided on the paper was accompanied by students' repeated visual orientations to it during their interactions. The fact that they were often looking at the paper rather than at Michelle, even when initially delivering the news of her husband's cardiac arrest, suggested that they did not have a primary orientation to the mutual visibility of the platform.

One common criticism of AI systems is that, in contrast with human actors, they focus only on language rather than action (Albert and Hall 2024). However, as our analysis shows, it seems that student participants in this simulation scenario often do the same. Rather than focus on the overall message they need to convey, they focus on repeating sections of the scenario text verbatim. As such, there is a lack of attention to the recipient design that is ordinarily a feature of interaction. One consequence is that unnecessary or overcomplex detail may be included, but the corollary is that this focus appears also to have an impact on what is left out. An obvious example is the way that the caller does not voluntarily identify the institution they are calling on behalf of, despite this being usual in institutional interactions. This information was not specifically given in the scenario paper, though all participants would have been familiar with local hospitals through their student placements and some drew on this in their interactions (e.g., in Extract 4 above). Although this information was absent when it would be interactionally relevant, it caused particular problems when it became interactionally *required*, as the example below illustrates:


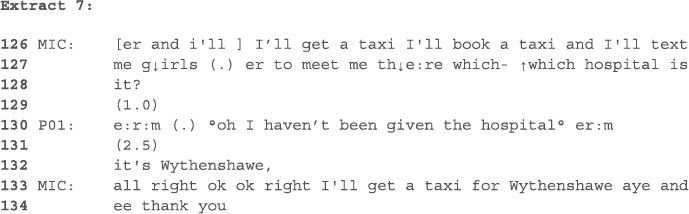


In this case, Michelle asks what hospital Dave is at; this is necessary information if she is to proceed with her proposal of booking a taxi to get there quickly (lines 127–128). There is a pause, and P01 temporarily breaks character from the simulation on line 130, prefaced by her ‘oh’ indicating a change of state (Heritage 1998). There is another longer pause, and she then names a local hospital. This situation is reminiscent of Stokoe et al.’s (2020) analysis of mystery shopper calls to veterinary practices, in which callers stumble when asked to produce hypothetical information (such as the weight of their imagined pet). However, in the simulation scenario here, both parties are aware that the situation is hypothetical, which perhaps explains why the break from character and pause are accepted by Michelle in line 133 without further interactional trouble.

In Albert and Hall's (2024) work on the use of virtual assistants such as Amazon's Alexa, they show how blaming a virtual assistant for a failed or incomplete action can fulfil a useful interactional function. In the example from the MHL simulation above, the participant also ascribes blame, in this case for their inability to respond quickly, to what they have ‘not been given’ in the paper scenario. However, in our data, this opportunity for blame extends beyond accounting for interactional delay; in other examples, student participants exploited the unreality of the scenario to attribute responsibility for any difficulties (e.g., in the availability of a bed on a cardiac ward to transfer Dave to) to others at the hospital. This is possible because, knowing the scenario is hypothetical, they do not have to be ultimately accountable for the truth of statements they make about what their imagined colleagues have or have not done, or will or will not do. However, this attribution of blame is in marked contrast to findings from research in hospital settings which show that HCPs do significant interactional work to avoid blaming their colleagues or even agreeing with patients' attributions of blame (Drewery 2023).

### 4.3 Progressivity/Lack of Urgency

If disordered (in the sense of nonnormative) openings and the management of shared knowledge represent two sites of interactional trouble, a third concerns the handling of urgency. Conversation analytic research across a range of everyday and institutional settings has shown that there is generally a preference for progressivity in human interaction (e.g., Slocombe et al. 2022; Stivers and Robinson 2006). As Mlynář et al. (2024) note, this preference persists even where virtual interactants are causing interactional trouble, so that the human participant carries the burden of sense‐making to achieve this progressivity. One specific criticism of large language model technologies in particular is that where urgency is a feature of interaction, they fail to identify, coconstruct and adapt to the urgency of the situation (Stokoe et al. 2024). The result is a further burden falling on the human participants. This burden is underpinned by the fact that CA research has shown that a person's stake in an encounter is fundamental to how that interaction proceeds (Edwards and Potter 1992). When there is no real patient and no real heart attack, there is no real urgency, and this is also reflected in the structure of the MHL interactions. We have already noted in the discussion of openings above that the reason for the call often comes late in these interactions (e.g., in line 14 in Extract 4 above). However, as in Extract 8, below, another significant way in which a lack of urgency manifests interactionally is in ‘reopenings’: moments when interactions could move to closure, but instead participants actively continue them.


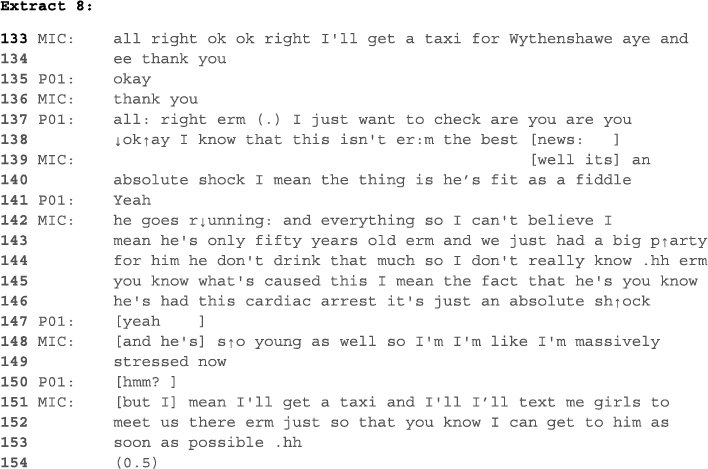


By this point in this interaction between Participant 01 and Michelle, the news of Dave's condition has been delivered and Michelle has here announced her intention to get a taxi to the hospital (line 133). Calling a taxi would necessitate ending this call and making another, and so this announcement creates a natural endpoint for the business of the call, where moving into closing sequences would be relevant. However, the student participant instead reopens the conversation on lines 137–138 by asking an open‐ended question about how Michelle is feeling. In response, Michelle begins her turn quickly, overlapping the end of P01's question (line 139). She expresses her shock, expanding to give a description of her assessment of Dave's good general health and of recent events that account for this shock. P01's response at line 150 appears to be a continuer, so it is Michelle who then returns to the practical action of getting a taxi to the hospital, explicitly topicalising the urgency of the situation and moving once again towards closure.

Extract 9 below also shows a reopening of the interaction at a point where a previous topic has concluded and so a transition to closing could be made. In this example, the student participant has told Michelle that her husband will be transferred to the cardiac intensive care unit, but by using the abbreviation ‘ICU’ as per the text in the scenario. She then goes on to explain what this means: that it is a more specialist setting for her husband's care.


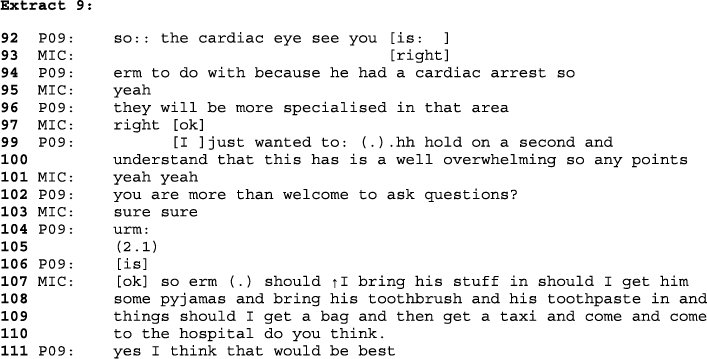


Michelle acknowledges the explanation provided about the move to ICU (‘right ok’ in line 97), and we then see a similar pattern occurring in which, rather than move to closing, the student topicalises Michelle's possible emotional state and uses this to offer an opportunity for further questions, thus reopening the interaction. Earlier in this interaction (in a section not shown here), Michelle has already expressed her feelings of shock; here, after an acknowledgement (line 103) and a pause, her questions are about the practical actions associated with travelling to the hospital.

Once again, this extract is reminiscent of Stokoe et al.’s (2020) findings in relation to mystery shoppers, and to questions of what stake participants have in the interaction. Both parties know that there is no real cardiac arrest here, and this lack of actual urgency is perhaps inevitably reflected in the structure of these closings. However, we suggest that these kinds of questions, about how Michelle is feeling, or whether she has any additional questions, also orient to the frequent use of these types of simulated scenarios as assessment tools for HCPs. Although our student nurse participants were informed that their performances were not being assessed, and that our interest was in the way interaction took place on the platform, the communication skills training that healthcare staff receive as part of their educational programmes points to the importance of soliciting unmet concerns and of attending to patient emotions (e.g., NHS England 2025; Jack 2025). In our data, news imparted to Michelle is initially formulated as urgent, but there is then a mismatch with this subsequent activity, which participants consider necessary to incorporate. Their conversational actions keep the conversation going in order to achieve this. The end result is that the descriptions of Dave's condition and the actions proposed for Michelle by the student participants speak to urgency, but their own interactional actions do not.

### 4.4 Closings

In the final section of our paper, we examine closings. As with openings, closings in these data were also disordered in the sense of departing from recognisable norms. One obvious example is that participants did not necessarily include a ‘goodbye’ sequence, sometimes simply terminating the call by clicking the ‘leave’ button, and sometimes explicitly demonstrating difficulties in knowing how to bring about an ending. Extract 10 is one such example of these difficulties:


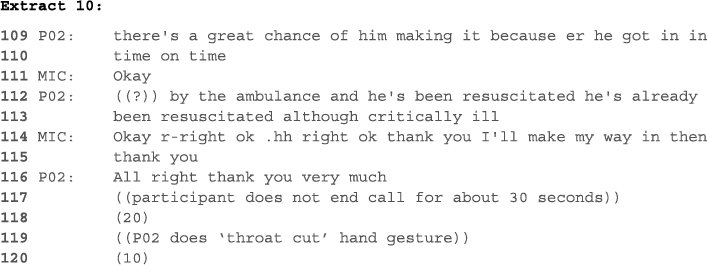


Here, the conversation reaches a preclosing point when agreement is reached that Michelle will make her way to the hospital (lines 114–116). However, after this point, there is no further talk, nor is the call ended. In the focus group discussion of student experiences of the simulation, participants spoke about their difficulties with knowing what should happen here. Some participants described expecting an automated ending, which is the case with some simulation platforms when all required activities have been completed, and is what appears to be expected in Extract 10. Again, this shows an orientation to these platforms as assessment tools, where ‘the system’, as some participants described it, judges the endpoint. Although other participants appeared to take closing cues from the interaction itself, terminal ‘goodbye’ sequences were not necessarily part of this, suggesting that even here the hybrid nature of MHL can be seen to have an impact. Extract 11 is a case in point:


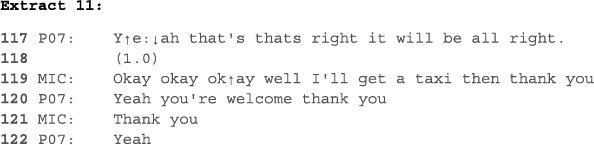


Here, after the closure of one topic and a pause signalling there is no move to a next topic. Instead, Michelle produces a series of preclosing ‘okays’ followed by a restatement of her previous proposal to get a taxi and an expression of thanks. These thanks are acknowledged in line 120 and repeated in line 121, but lead to a ‘yeah’ rather than a goodbye from the student participant, followed by a termination of the call.

Mlynář et al.’s review of existing literature (2024) shows a variety of ways of ending an interaction with a robot, including leaving the interaction without doing a closing at all (e.g., by walking away). They suggest that how closings are done is reflective of the system's perceived status in the interaction and of whether technology is treated as an interactional partner or an object. Our data show that, despite the claimed realism of the MHL platform, there remains an ambiguity here for participants. This is the case even though some members of the focus group reported recognising the voice of the tutor live‐linked to Michelle, and therefore had personal knowledge of the human producing the interaction.

## 5 Discussion

Our analysis above has shown how openings in these hybrid simulations were routinely disordered relative to the norms documented in typical call openings. Student callers typically spoke first and often had to redo opening sequences. Secondly, we showed how the scenario brief supplied a ‘script’ that sometimes overpowered the situated interactional management of shared knowledge. For example, students would use words and phrases from the script verbatim rather than designing their turns for the displayed interactional needs of their recipient. Thirdly, we observed that the interactions reflected ill‐fitted ‘stakes’ and a lack of urgency given the simulated scenario, for example, where ‘reopenings’ deferred closings and next actions that were obviously time‐sensitive. Finally, we also saw that closings were often truncated or absent. We now consider the significance of these findings.

Our findings echo some previous work on technologically mediated interaction. They also show similarities with research into telephone ‘mystery shopping’, where the participant interacting as a client does not have the same stake in an interaction that an authentic client would, yet ‘passing’ (in Garfinkel's (1967) sense of achieving the desired status of) as authentic requires a practical display of this stake and interest (Stokoe et al. 2020). However, none of these previously researched scenarios maps directly to the hybrid nature of MHL, and what we have shown here is that the proposed improved realism that the use of this system offers can actually create new interactional difficulties and ambiguities. The end result is that it is harder than might be imagined to accomplish what Stokoe et al. (2020, 91) call ‘the job of being an authentic party to an interaction’.

In particular, the epistemics of who is to be treated as knowing what is particularly complex, leading to student participants delivering information to Michelle with a clear lack of orientation to recipient design. This manifests both in the giving of inappropriate information that someone would inevitably already know about their partner, as well as unnecessary or unintelligible details such as exact timings of events or technical medical terms. We suggest that these epistemic issues are, in part, explained by an orientation to clinical rather than interactional authenticity, and to assessment rather than conversation. Specifically, these aspects of interactions are produced through a focus on the transmission of information exactly as it was given in the scenario outline, including the repetition of exact terms or phrases that were contained there even when these were problematised by the recipient. Albert and Hall (2024) have described how AI‐mediated systems generally focus on language rather than action, but it seems in this context that the student participants also do the same.

This focus on language rather than action also relates to our findings around progressivity, so that, whereas participants' words may suggest urgency, their actions (such as reopening of interactions) do not match this. Stokoe et al. (2020, 106) have suggested the application of a ‘conversation analytic Turing test’, which asks whether simulated interaction yields ‘functionally equivalent social actions and, thereby, similar social relationships’. Our analysis here shows the limitations of this particular mode of interaction in this respect, in the sense that participants can successfully simulate language use, but do not manage to coconstruct a situation that creates action in the same way an actual interaction involving the cardiac arrest of a spouse would.

Previous research (e.g., Atkins 2019) has suggested a distinction between the use of simulation for teaching and training versus its use for assessment, where the former is seen as less problematic than the latter. However, our findings suggest that it can be hard for learners to make this distinction in practice. Because students are aware these platforms *are* used for assessment, and have experience of them being used in this way, our analysis suggests they orient to these interactions as assessments. This manifests in participants using their knowledge of features that are commonly assessed to navigate the interaction. For example, the way learners reopen interactions to enquire after the psychological health of Michelle, or to ask whether she has any additional concerns, would delay her hypothetically urgent arrival at the hospital. This suggests that learners are concerned with performing the commonly assessed practice of eliciting feelings rather than achieving the healthcare outcome the simulation is designed to support.

Our findings, taken together, point to specific contributions and implications of this study. Whereas previous conversation analytic work has examined role play with actors or fully automated agents, the hybrid MetaHuman platform combines a live human voice and gaze with a synthetic, reconfigurable appearance. Here we have shown that this combination does not straightforwardly increase interactional authenticity. Indeed, we have shown how this configuration can generate its own ambiguities and interactional problems for learners. This finding has practical implications for educators. It suggests that the interactional artifice of such platforms may be better incorporated as an integral and explicit part of the teaching and learning process, rather than being technologically ‘designed away’. It also points to the risk that simulation designs using complex technological systems, task instructions and briefing materials introduce additional interactional resources that students orient to, sometimes at the expense of recipient design and the ostensible purpose of the training. Compared with actor‐based simulation, hybrid systems may offer scalability and flexibility, but our analysis cautions against assuming that enhanced visual realism necessarily results in more interactionally authentic simulated encounters.

Our study also has limitations. It is based on a single‐site sample of ten simulated interactions involving only one scenario and one simulator, and we make no claim to generalisability. The empirical value of these analyses lies in what they reveal about the interactional organisation of hybrid simulation, and how they compare with results from the last 70 years of conversation analytic research, which is likely to be transferable to similar platforms and design choices as the use of such platforms expands. Further research with larger and more varied populations, clinical scenarios and simulation platforms with a range of design principles would help to establish how transferable these patterns are.

## 6 Conclusions

Here we have presented an analysis of simulated interactions in a hybrid human/computer‐mediated setting, using an avatar mapped onto the talk and movements of a human simulator. Our analytical method has demonstrated the sociological value of CA for identifying and describing the interactional detail of simulations in ways that provide actionable insights. As Brandt et al. (2024) have previously noted, this critical approach can help to guide system design. Similarly, understanding the strengths and limitations of the MHL platform and its relationship to concepts of ‘interactional authenticity’ can reveal how hybrid human/computer simulations can be most effectively integrated into communication skills training for HCPs. We therefore propose two possible approaches to these kinds of hybrid systems. One is to develop simulation scenarios based on the actual interactional practices that participants are shown to use in a particular healthcare setting, and to train ‘patient’ participants to use these practices rather than their imagined ideas of what someone in this scenario *might* say (Pilnick et al. 2023). However, although this approach has been shown to enhance interactional authenticity in the sense that trainees deal with actions and practices that actually occur in real‐life settings, it can be costly and time‐consuming and is unlikely to be feasible in all areas of clinical practice. In addition, although grounding simulated scenarios in evidence of real interactional practices can improve authenticity, we would not want to suggest that this authenticity can be continually incrementally engineered. Our analysis suggests that the difference between simulated and real interactions is constitutive—not simply a deficit of fidelity to be closed. We therefore advance the methodological and study‐design approach taken here cautiously, as a means of grounding and evaluating simulation scenarios in observed practices, rather than implicitly claiming that simulations might be rendered as fully ‘authentic’.

The second is to acknowledge, at the outset, that although simulated and technologically mediated interaction in healthcare can be valuable educational tools, they are not a direct substitute for face‐to‐face human interaction. Previous research by Bartram (2020) and Iedema et al. (2009) has suggested that these differences can offer pedagogical spaces, but this line of argument does not easily translate into a context where interaction itself is the primary learning focus. Identifying and explicating interactional reasons for differences in technologically mediated interactions provides an opportunity to draw users' attention to the interactional artificiality of simulation, and to do so in ways that encourage reflection on what this means for both their healthcare training and practice.

These two alternative approaches lead us to consider the wider sociological ramifications of these kinds of technologically mediated simulations. The problem with technologies that emulate human interaction is not located purely within the technologies themselves. Focusing on how ‘authenticity’ can be improved may be important for practical utility, but it is ultimately a solutionist approach, which risks distracting from the fact that the sociological project to explicate how talk‐in‐interaction works in practice is far from complete. It follows that we cannot technologically emulate, or simulate, something that we do not fully understand. As we enter an age in which the means and methods of interaction continue to expand, Goffman's (1983) call for sociologists to treat the interaction order as a substantive domain for study in its own right remains as pertinent as ever. Several questions remain for future research. What forms of interactional competence should learners be helped to develop when their ‘recipient’ is a hybrid agent? And how might briefing materials be redesigned to enhance, rather than replace, recipient design? How do the issues identified here recur across more diverse clinical and simulated settings, with different platforms and among participants with different training and experience? And how should the interaction order of simulated AI‐mediated encounters be theorised as a domain that is neither straightforwardly ‘real’ nor simply ‘artificial’? Addressing these questions will require the kind of close, comparative studies of situated interaction that conversation analysis, and sociology more broadly, is well positioned to provide.

## Author Contributions

**Alison Pilnick:** conceptualisation, data curation, formal analysis, methodology, investigation, visualisation, project administration, supervision, writing – original draft, funding acquisition. **Lauren Bridgstock:** data curation, formal analysis, methodology, investigation, project administration, visualisation, writing – original draft. **Saul Albert:** funding acquisition, formal analysis, methodology, writing – review and editing. **Rebecca O'Brien:** funding acquisition, writing – review and editing, formal analysis, methodology. **Anne‐Marie Borneuf:** funding acquisition, writing – review and editing, investigation, methodology, conceptualisation.

## Funding

This research was funded by a Research Development Award from the Foundation for the Sociology of Health and Illness.

## Ethics Statement

This research was approved by the Manchester Metropolitan University Faculty of Health and Education Research Committee.

## Conflicts of Interest

The authors declare no conflicts of interest.

## Data Availability

Because the dataset comprises unanonymised video data, conditions of ethical approval and best practice guidance from the UK Data Archive mean it is not available for sharing.

## Endnotes

^1^
Participant pseudonyms were created by asking Microsoft Copilot to suggest names with the right number of syllables, matching gender and similar regional and cultural backgrounds.

^2^
Although ‘cardiac arrest’ is a commonly used term in healthcare, the British Heart Foundation charity suggests it is not widely understood in the UK and is often wrongly assumed to be an exact synonym for ‘heart attack’ https://www.bhf.org.uk/informationsupport/conditions/cardiac‐arrest.
